# Supplementary material for: Development of a Tandem Mass Spectral Library for the Detection of Triterpenoids in Plant Metabolome Based on Reference Standards
Source: Plants (Basel). 2024 Nov 22;13(23):3278. doi: 10.3390/plants13233278 (PMC11644824; doi:10.3390/plants13233278)
Supplement: Supplementary file 1 [file plants-13-03278-s001.zip › plants-3324720-supplementary.pdf]

SUPPORTING INFORMATION

Development of Tandem Mass Spectral Library for the Detection of Triterpenoids in Plant Metabolome based on Reference Standards

Bibi Zareena [1], Syed Usama Y. Jeelani [1], Adeeba Khadim [1], Arslan Ali [2], Jalal Uddin [3], Satyajit D. Sarker [4], Matthias Rainer [5], Hesham El-Seedi [6,*], Muhammad Ramzan [1], Syed Ghulam Musharraf [1,2,*]

[1] H.E.J. Research Institute of Chemistry, International Center for Chemical and Biological Sciences, University of Karachi, Karachi-75270, Pakistan. zarina_ku@yahoo.com; syedusamayaseen@gmail.com; adeeba.abbas@gmail.com; rafridi73@gmail.com

[2] Dr. Panjwani Center for Molecular Medicine and Drug Research, International Center for Chemical and Biological Sciences, University of Karachi, Karachi-75270, Pakistan. arslanali1986@gmail.com

[3] Department of Pharmaceutical Chemistry, Colleg of Pharmacy, King Khalid University, Asir 61421, Saudi Arabia. jalaluddinamin@gmail.com

[4] School of Pharmacy and Biomolecular Sciences, Liverpool John Moores University, James Parsons Building Byrom Street, Liverpool, L3 3AF, United Kingdom.

[5] Institute for Analytical Chemistry and Radiochemistry, University of Innsbruck, A-6020 Innsbruck, Austria. m.rainer@uibk.ac.at

[6] Department of Medicinal Chemistry, Uppsala Biomedical Center, Uppsala University, Sweden. hesham.el-seedi@fkog.uu.se

* Correspondence: musharraf1977@yahoo.com; Tel.: (+92 21 34824924-5; 4819010)

Table of Contents

Table S1: All Standard triterpenoids sstructures, classes and MS/MS spectra in the constructed Library

Table S1: All Standard triterpenoids structures, classes, and MS/MS spectra in the constructed Library

S.No.	Name (Barcode)	Subclass of Compounds	Structure/Exact Mass/Chemical Formula	Spectra in constructed library
1.	Betulinic acid (STD005)	lupane	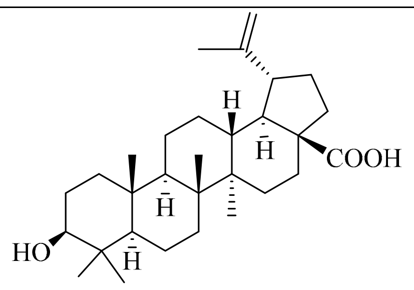 Chemical Formula: $C_{30}H_{48}O_3$ Exact Mass: 456.36	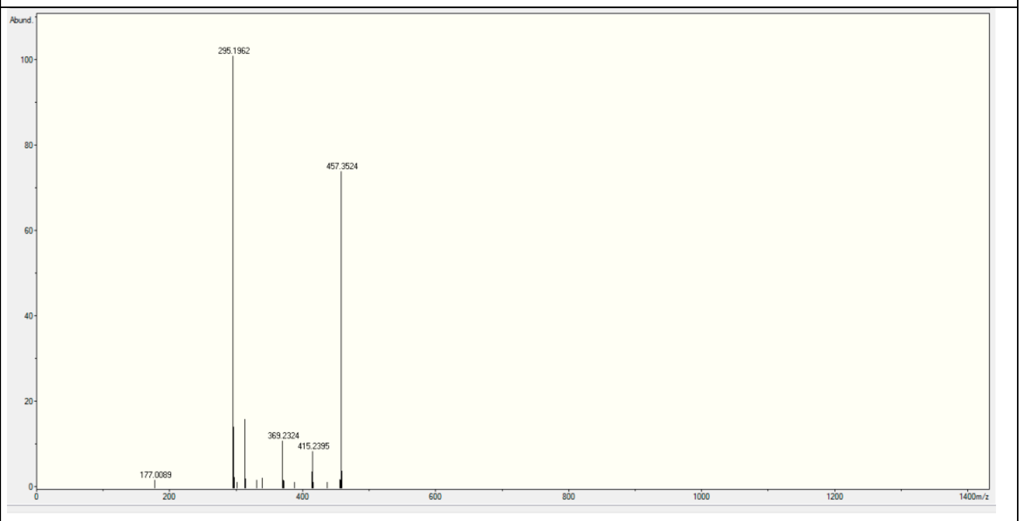
2.	Betulin (STD004)	lupane	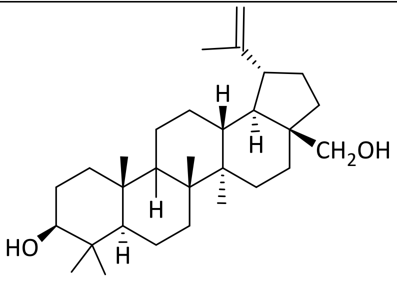 Chemical Formula: $C_{30}H_{50}O_2$ Exact Mass: 442.3811	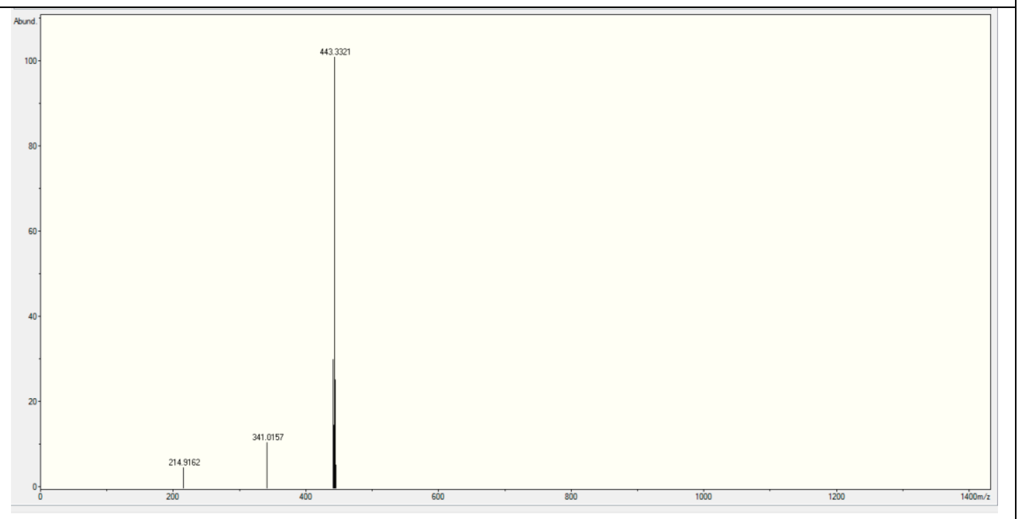
3.	Ursolic acid (STD007)	Ursane	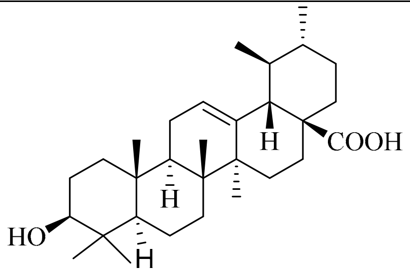 Chemical Formula: $C_{30}H_{48}O_3$ Exact Mass: 456.3603	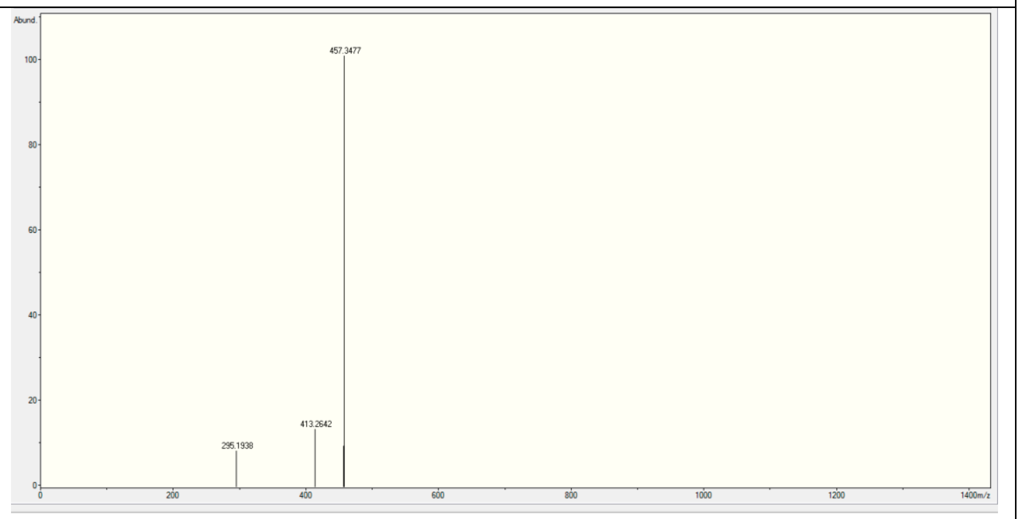
4.	Oleanolic Acid (STD011)	Oleanane	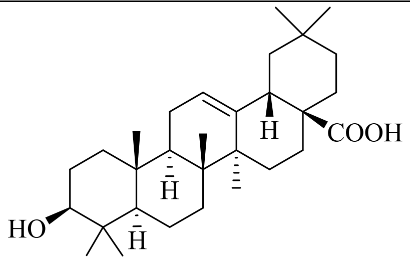 Chemical Formula: $C_{30}H_{48}O_3$ Exact Mass: 456.3603	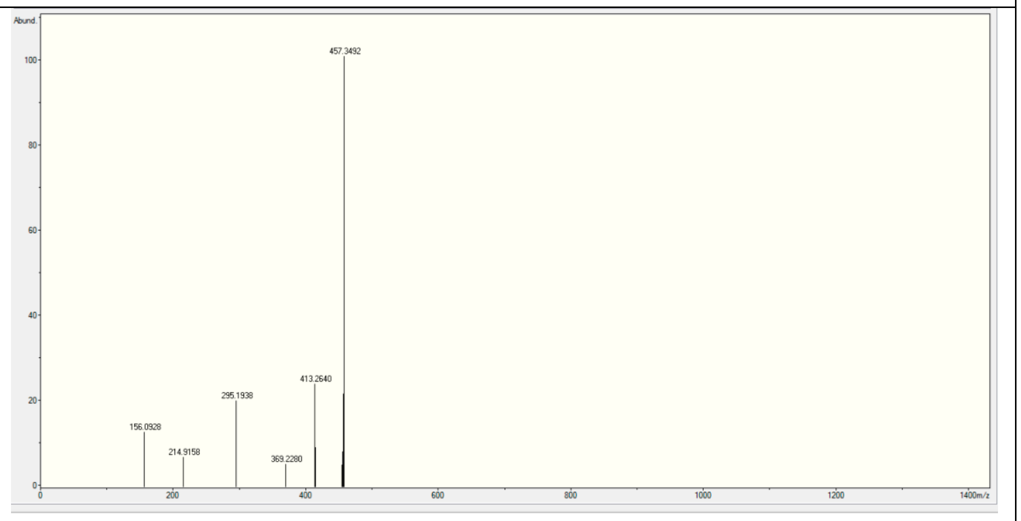

5.	Silymin A<br>(AAC139)	Ursane	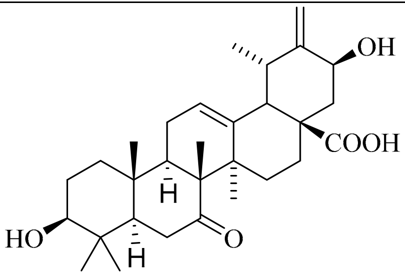<br>Chemical Formula: $C_{30}H_{44}O_5$<br>Exact Mass: 484.3189	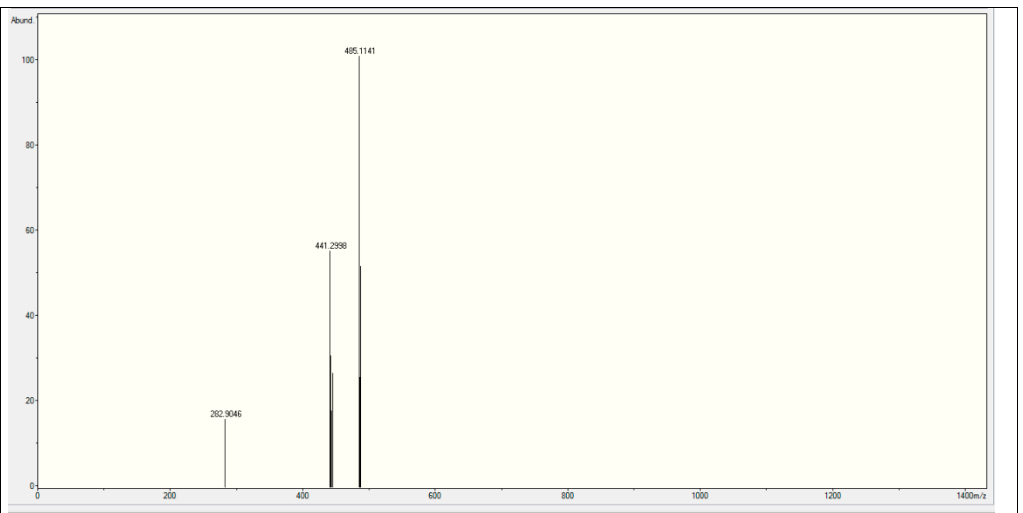
6.	Intybusoloid<br>(AAC169)	Limonoid	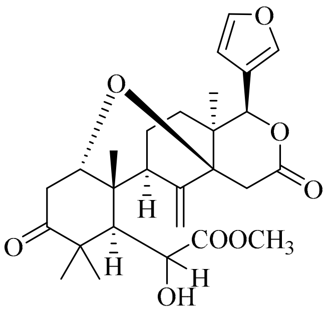<br>Chemical Formula: $C_{27}H_{34}O_8$<br>Exact Mass: 486.2254	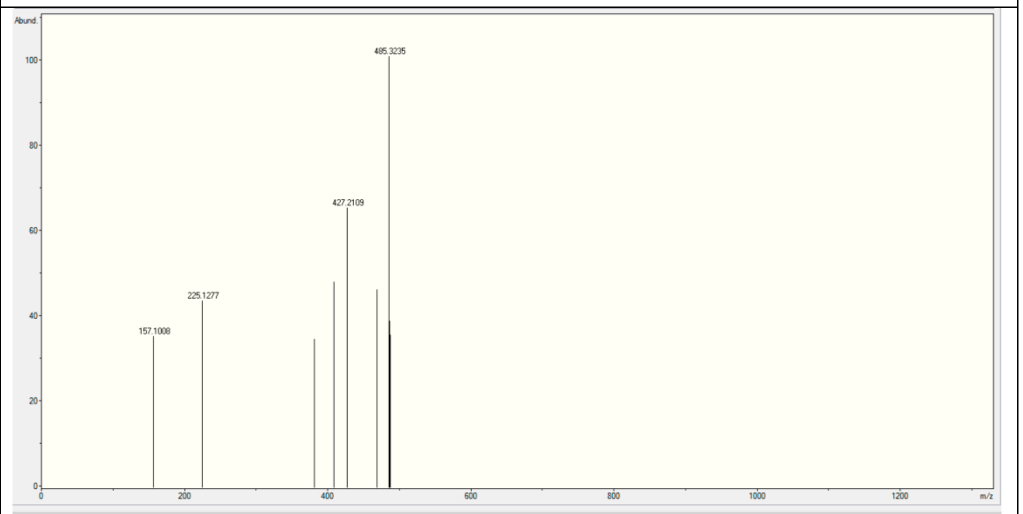
7.	Bellerigenin B<br>(AAC192)	Oleanane	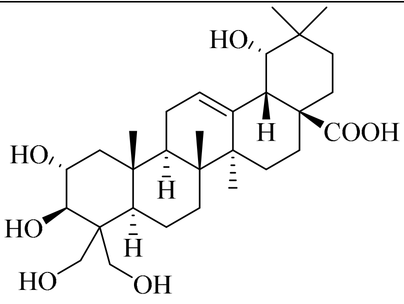<br>Chemical Formula: $C_{30}H_{48}O_7$<br>Exact Mass: 520.3400	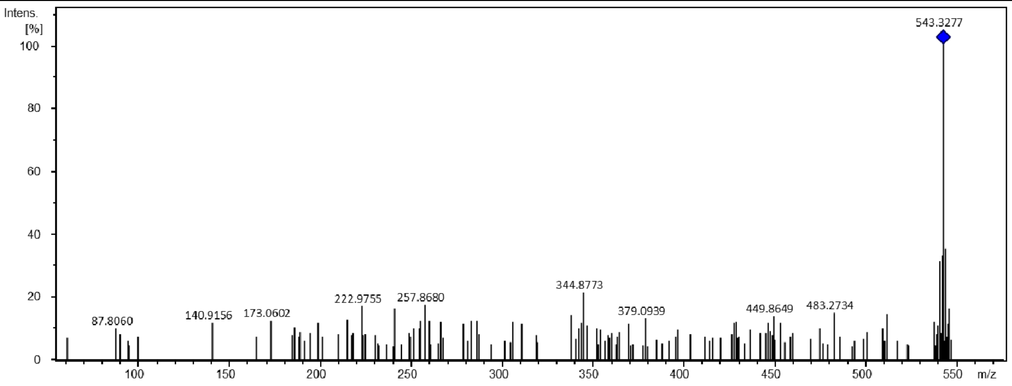
8.	ilelatifol D<br>(AAC196)	Ursane	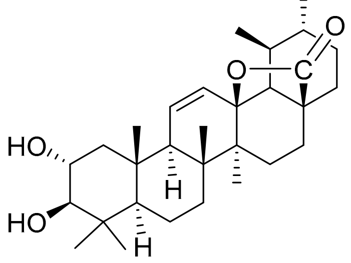<br>Chemical Formula: $C_{30}H_{46}O_4$<br>Exact Mass: 470.3396	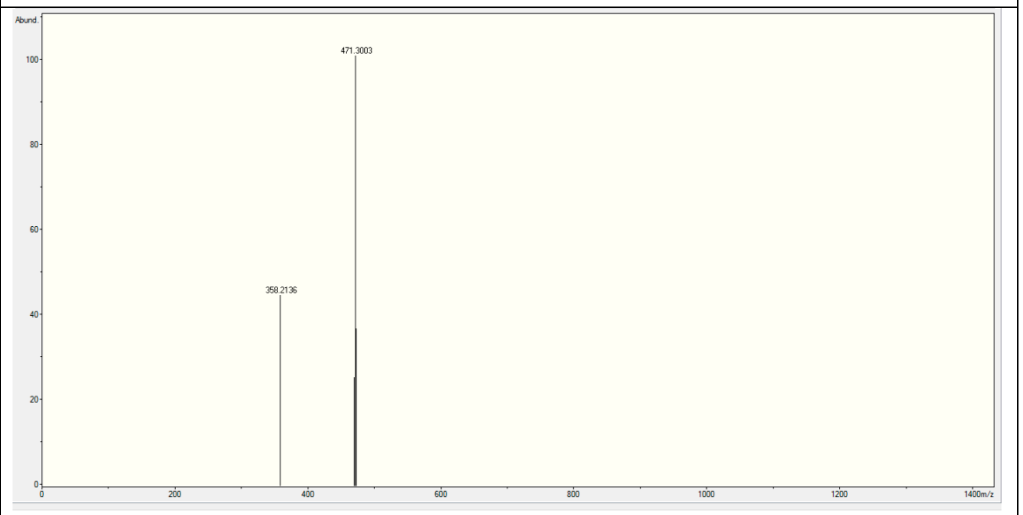
9.	Euscaphic acid<br>(AAC198)	Ursane	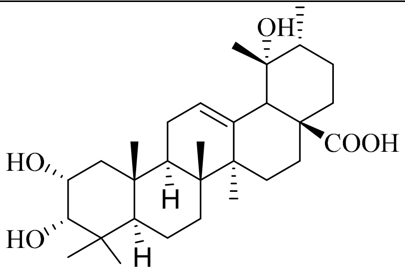<br>Chemical Formula: $C_{30}H_{48}O_5$<br>Exact Mass: 488.3502	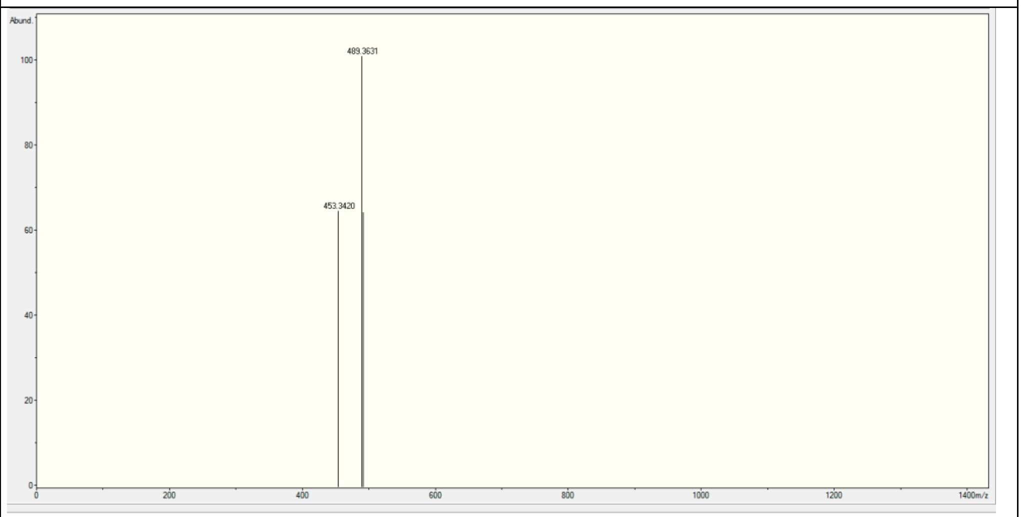

10.	Glycyrrhetic acid (AAC411)	oleanane	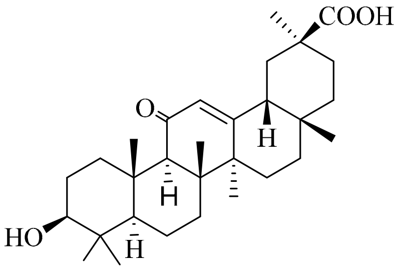<br>Chemical Formula: $C_{30}H_{46}O_4$<br>Exact Mass: 470.3396	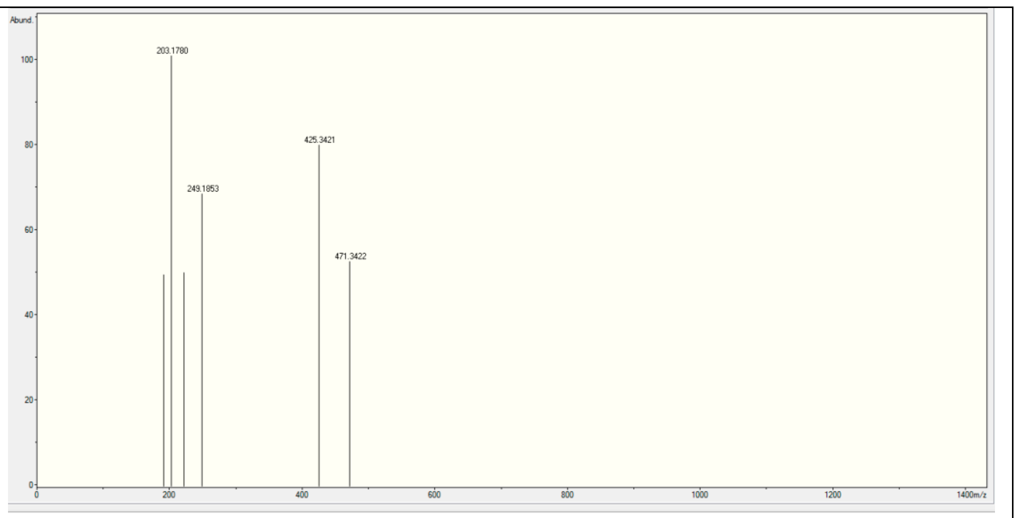
11.	Oleanoic acid (AAC451)	Oleanane	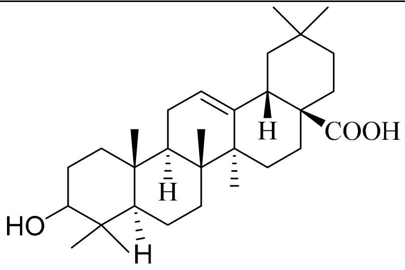<br>Chemical Formula: $C_{30}H_{48}O_3$<br>Exact Mass: 456.3603	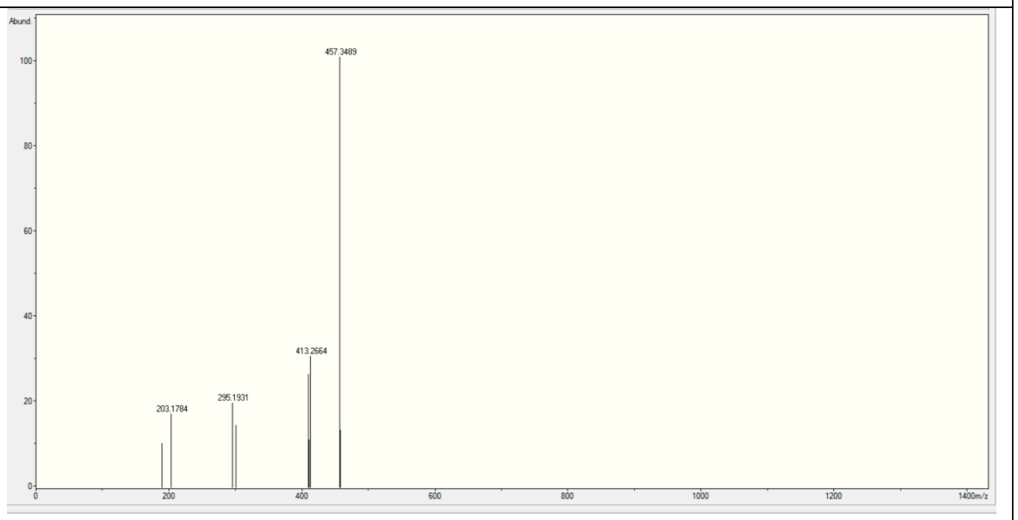
12.	Ursonic acid (AAC452)	Ursane	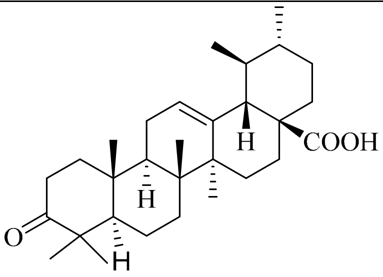<br>Chemical Formula: $C_{30}H_{46}O_3$<br>Exact Mass: 454.3447	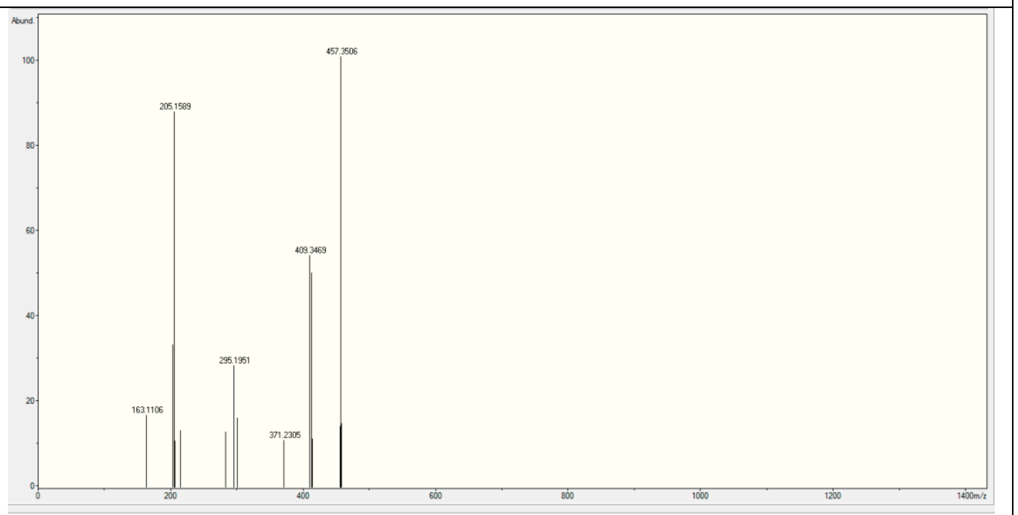
13.	Lantanilic acid (AAC456)	Oleanane	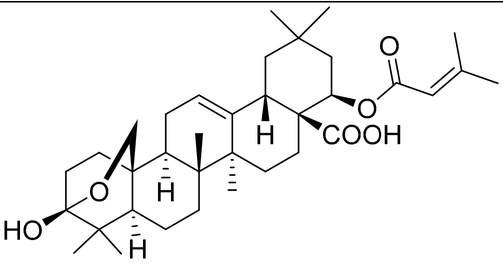<br>Chemical Formula: $C_{35}H_{52}O_6$<br>Exact Mass: 568.3764	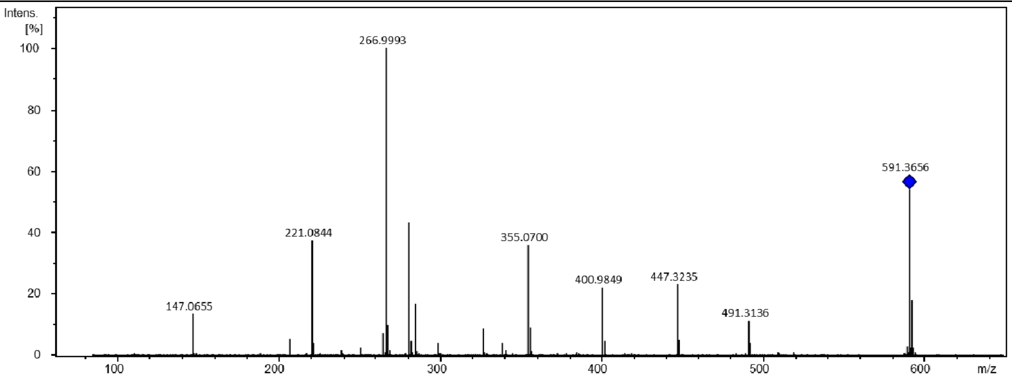
14.	3,25-epoxy-3a,hydroxy-olean-12-en-28-oic acid (AAC457)	Oleanane	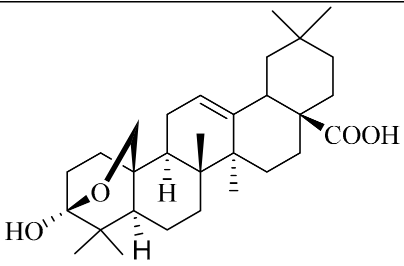<br>Chemical Formula: $C_{30}H_{46}O_4$<br>Exact Mass: 470.3396	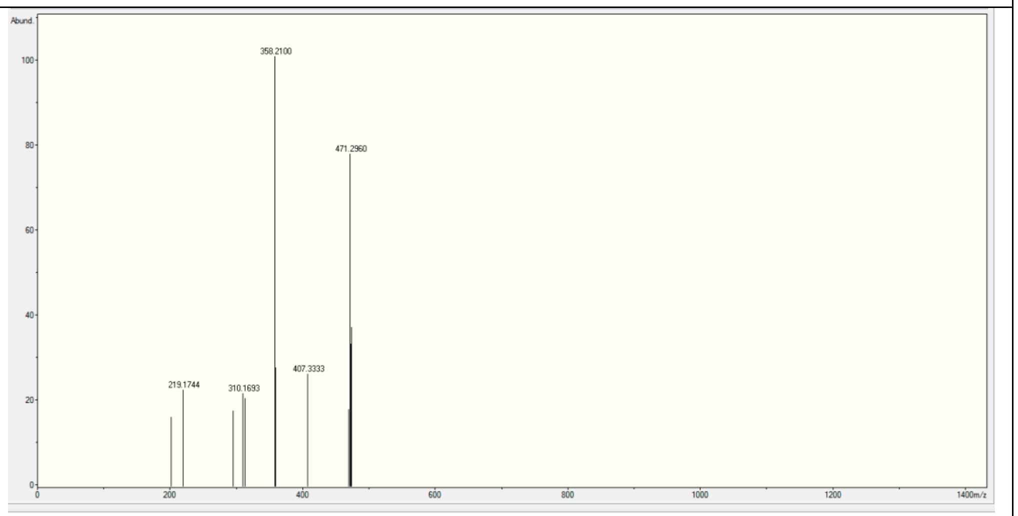

15.	2-a hydroxyurasolic acid<br>(AAC515)	Ursane	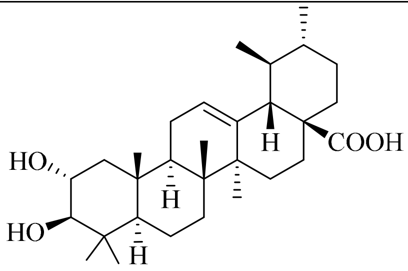<br>Chemical Formula: $C_{30}H_{48}O_4$<br>Exact Mass: 472.3553	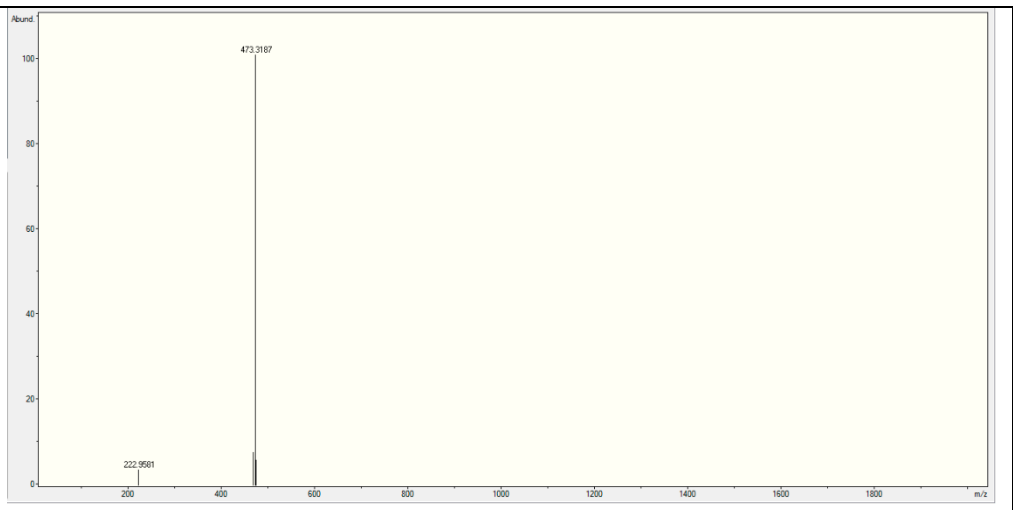
16.	Nimbinolide<br>(AAC690)	Limonoid	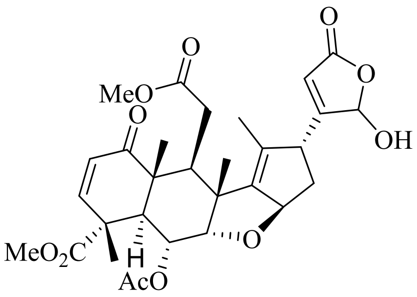<br>Chemical Formula: $C_{30}H_{36}O_{11}$<br>Exact Mass: 572.2258	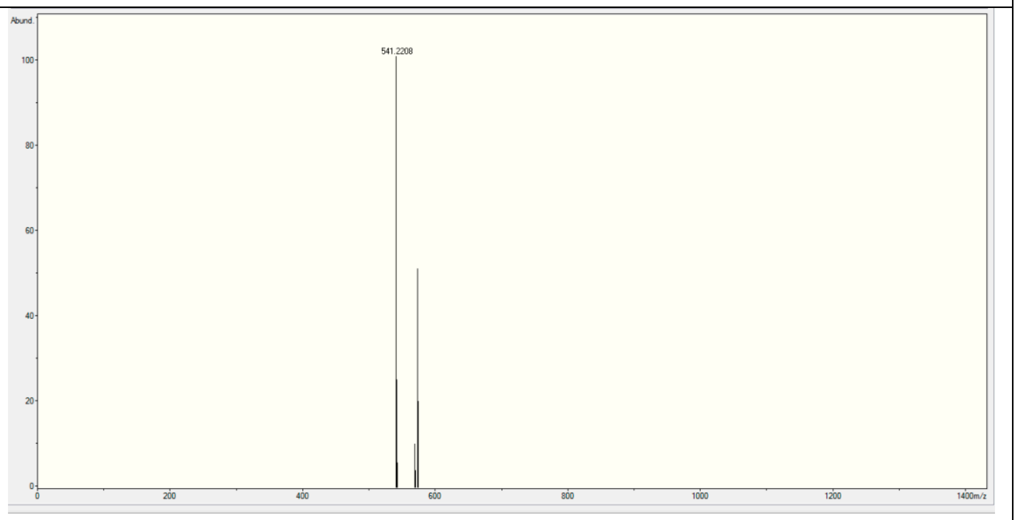
17.	Gedunin<br>(AAC691)	Limonoid	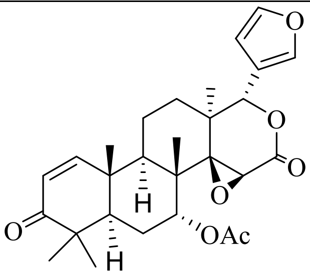<br>Chemical Formula: $C_{28}H_{34}O_7$<br>Exact Mass: 482.2305	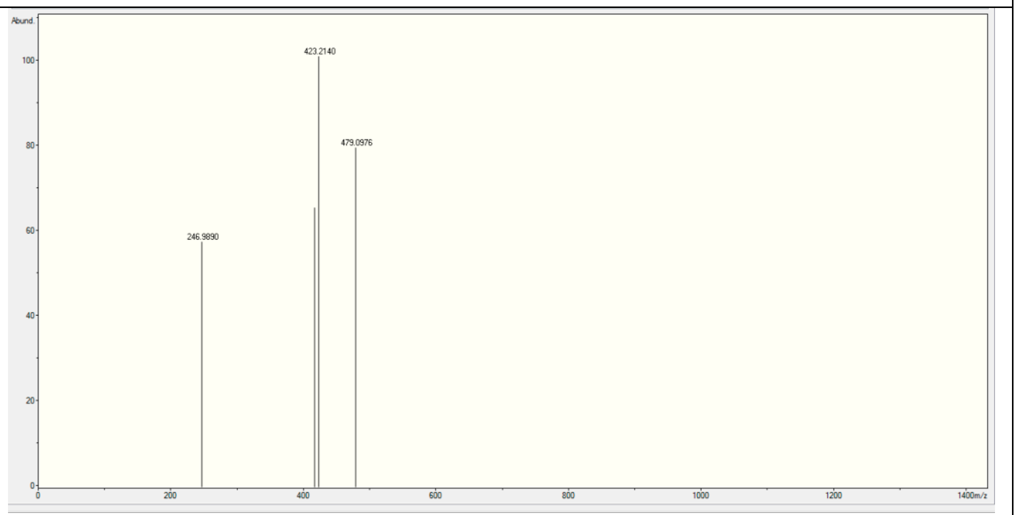
18.	Azadiradione<br>(AAC694)	Limonoid	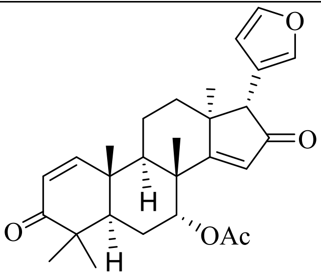<br>Chemical Formula: $C_{28}H_{34}O_5$<br>Exact Mass: 450.2406	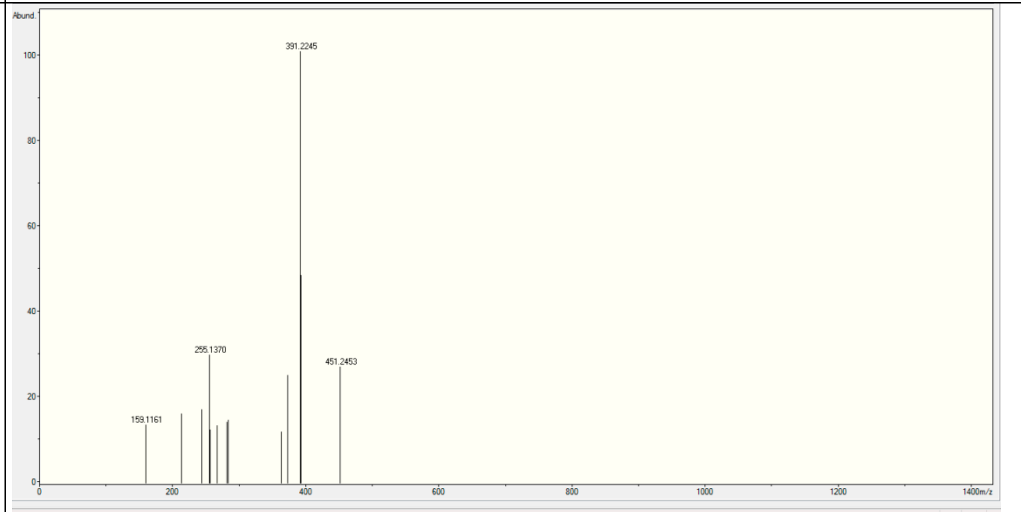
19.	3-Oxolup-1:12-diene, 28-al<br>(AAC715)	Lupane	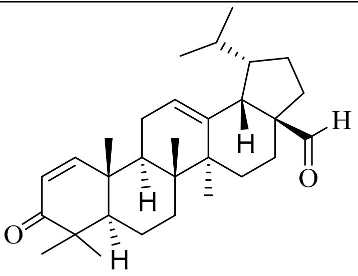<br>Chemical Formula: $C_{30}H_{44}O_2$<br>Exact Mass: 436.3341	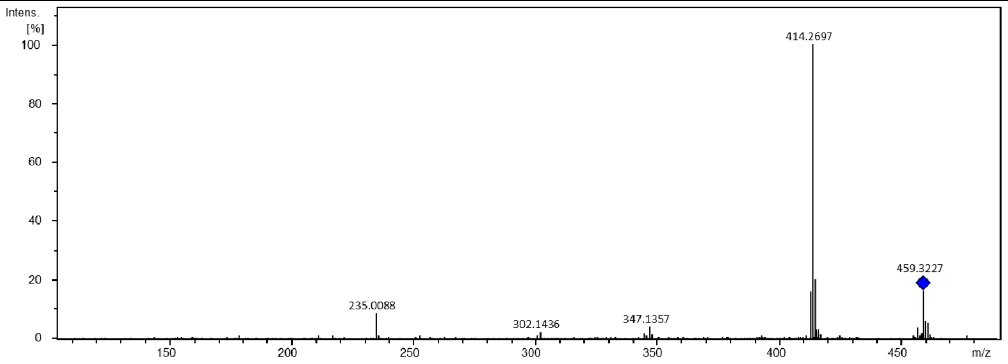

20.	β -Neriursate (AAC733)	Ursane	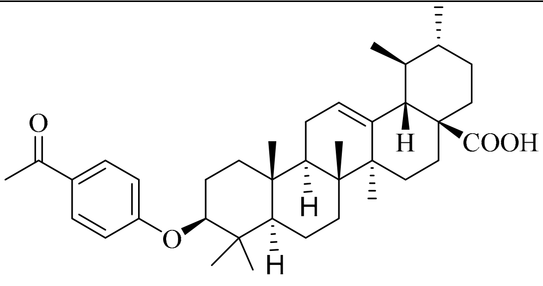 Chemical Formula: $C_{38}H_{54}O_4$ Exact Mass: 574.4022	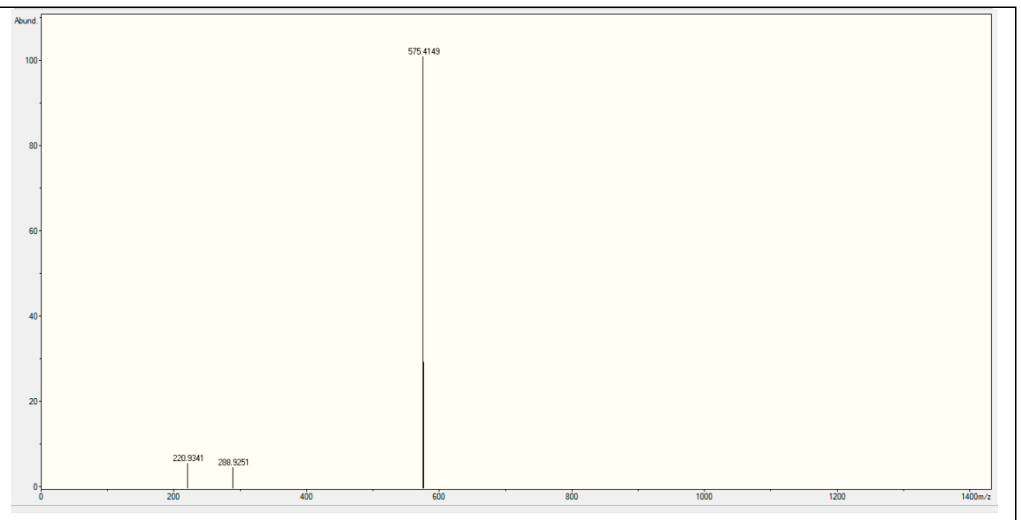
21.	Ursolaldehyde (AAC738)	Ursane	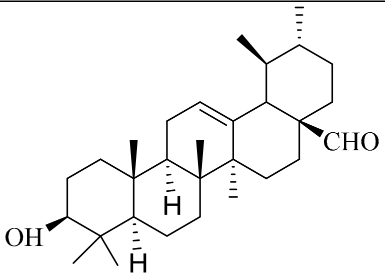 Chemical Formula: $C_{30}H_{48}O_2$ Exact Mass: 440.3654	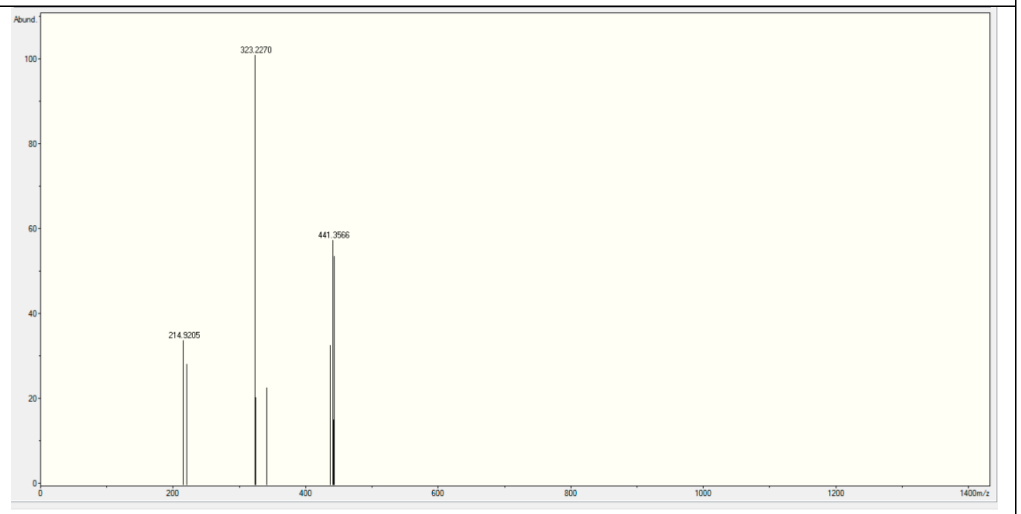
22.	3b-Hydroxyurs-11-en-13b(28)-olide (AAK089)	Ursane	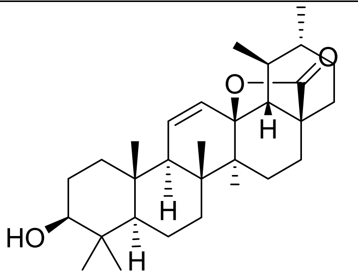 Chemical Formula: C30H46O3 Exact Mass: 454.3447	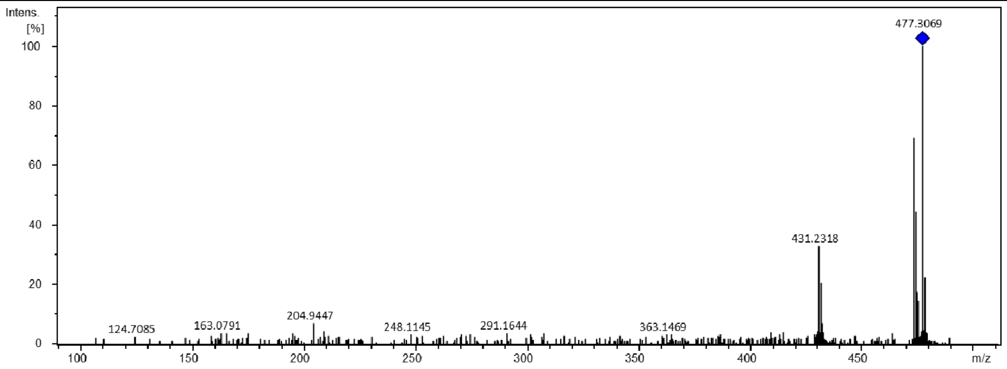
23.	11-Oxooleanolic acid (AAK094)	oleanane	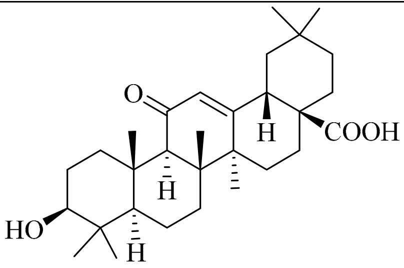 Chemical Formula: $C_{30}H_{46}O_4$ Exact Mass: 470.3396	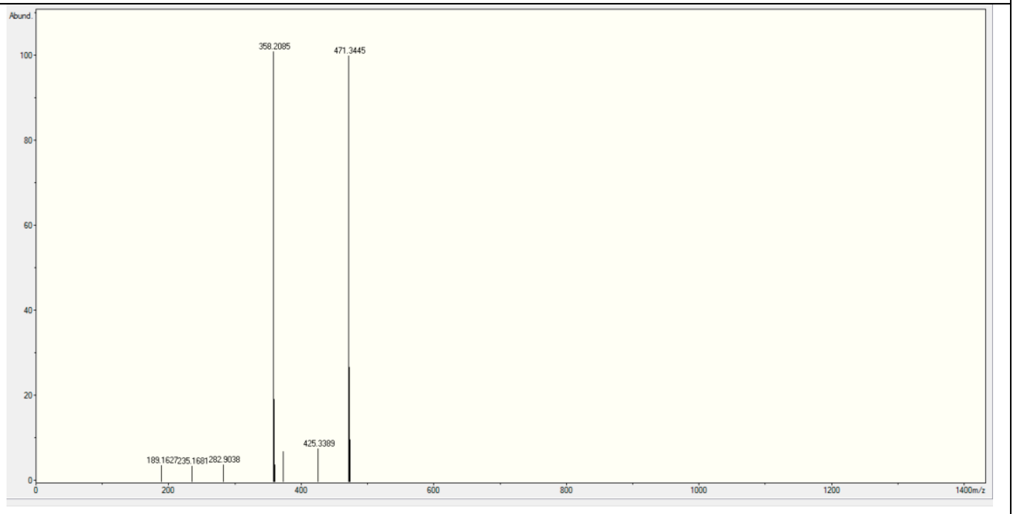
24.	5-hydroxy-7-{{6-O-{[(4R/S)-4-(1-hydroxy-1-methylethyl)cyclohex-1-en-1-yl]-carbonyl}-D-glucopyranosyl}oxy}-2-methyl-4H-1-benzopyran-4-one (AAC039)		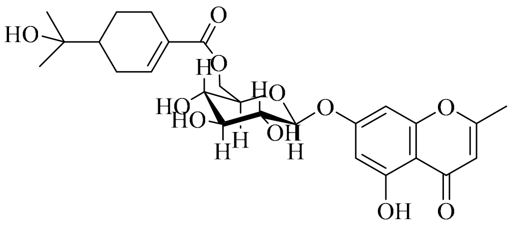 Chemical Formula: $C_{26}H_{32}O_{11}$ Exact Mass: 520.1945	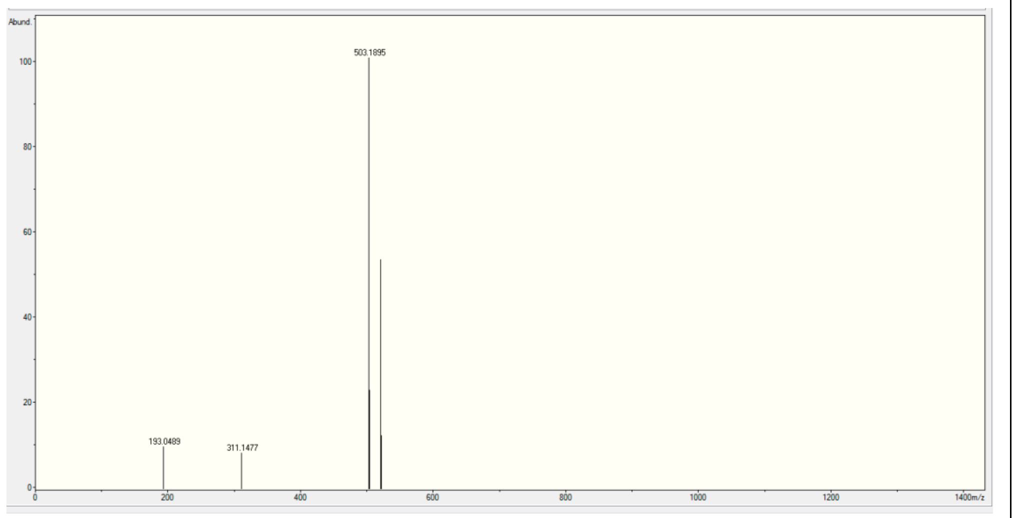

25.	Asiatic acid<br>(AAK121)	Ursane	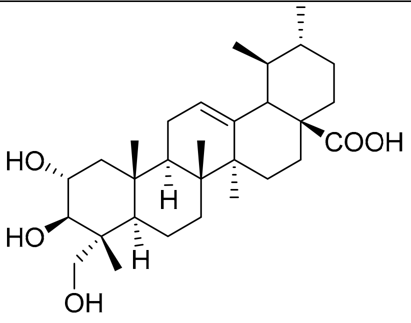<br>Chemical Formula: $C_{30}H_{48}O_5$<br>Exact Mass: 488.3502	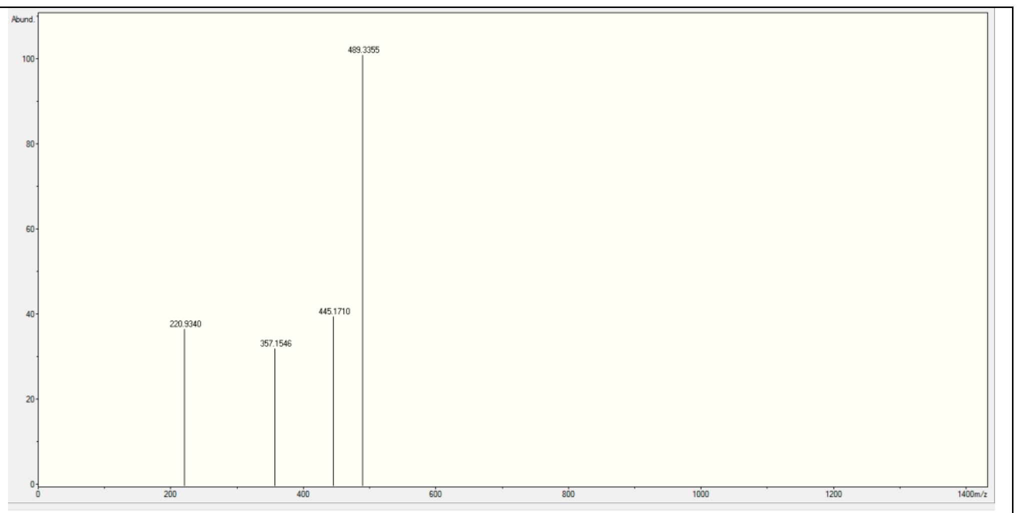
26.	Butyl ester of glycyrrhetinic acid<br>(AAK129)	Oleanane	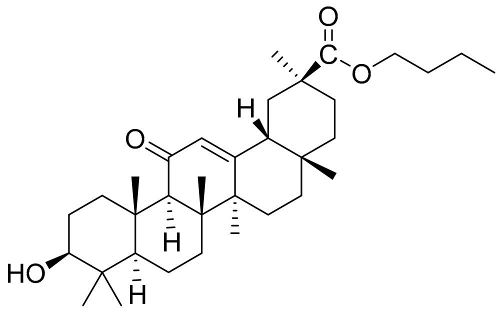<br>Chemical Formula: $C_{34}H_{54}O_4$<br>Exact Mass: 526.4022	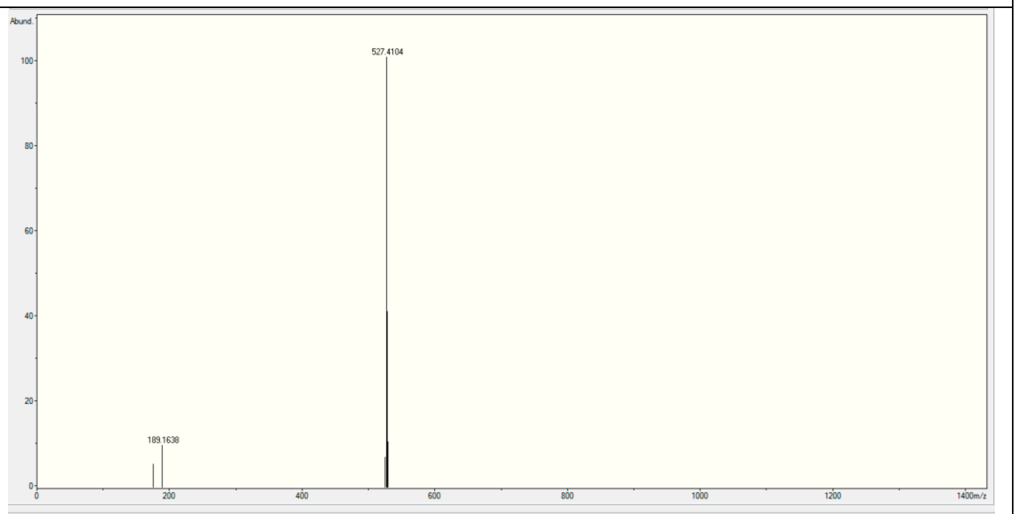
27.	3-oxo-30-butyl ester of glycyrrhetinic acid<br>(AAK130)	oleanane	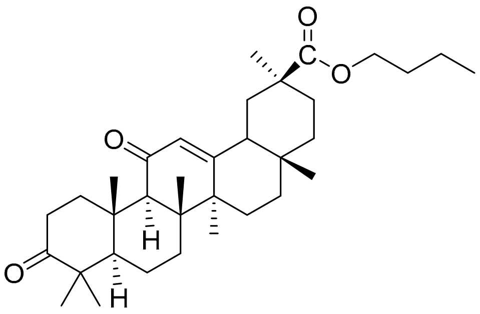<br>Chemical Formula: $C_{34}H_{52}O_4$<br>Exact Mass: 524.3866	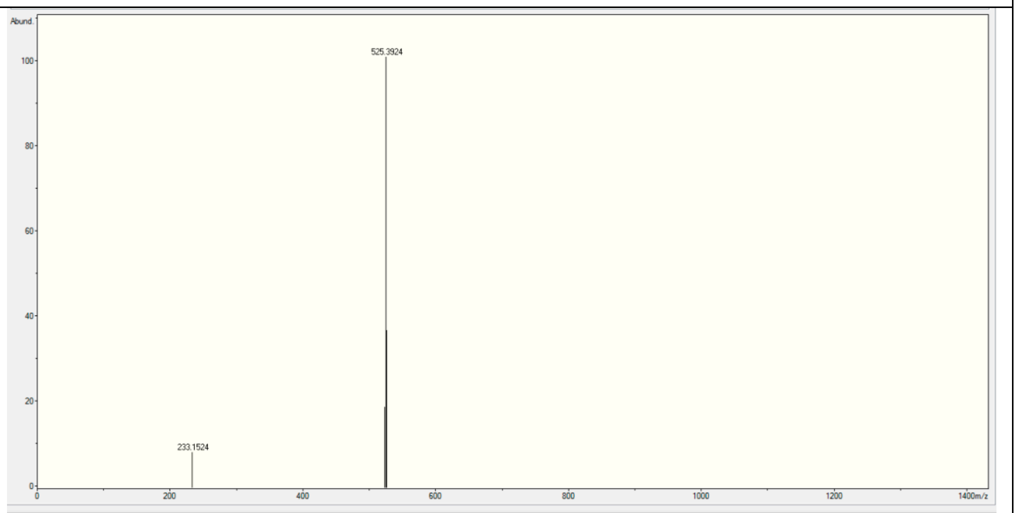
28.	Glycyrrhetinic acid methyl ester<br>(AAK131)	oleanane	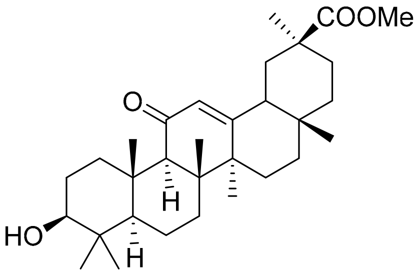<br>Chemical Formula: $C_{31}H_{48}O_4$<br>Exact Mass: 484.3553	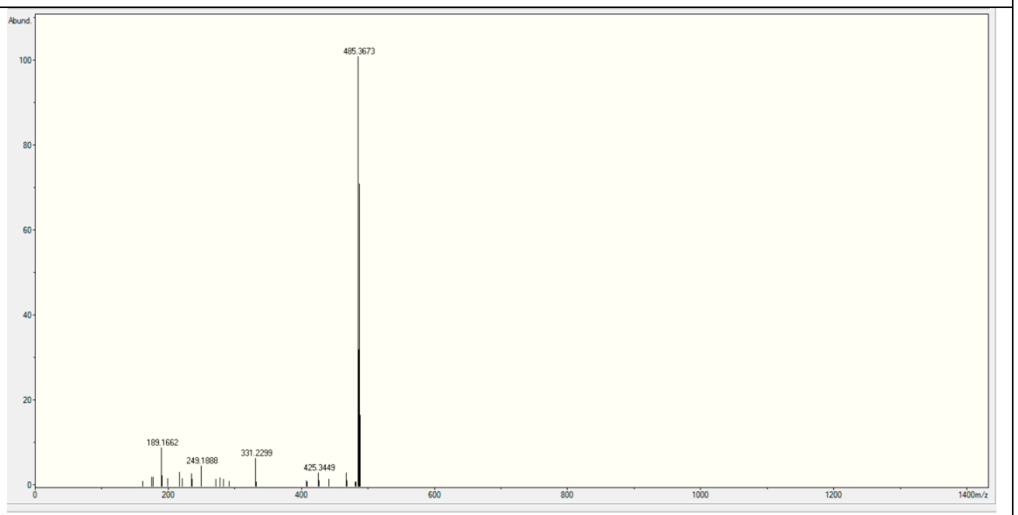
29.	3-benzoyloxy-3-0-methyl ester of glycyrrhetinic acid<br>(AAK132)	oleanane	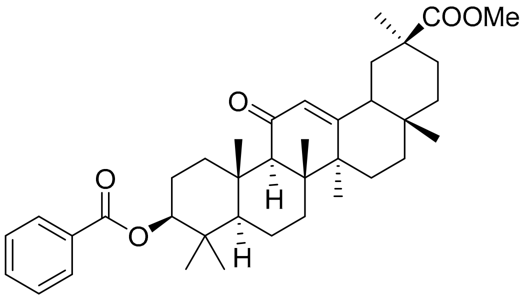<br>Chemical Formula: $C_{38}H_{52}O_5$<br>Exact Mass: 588.3815	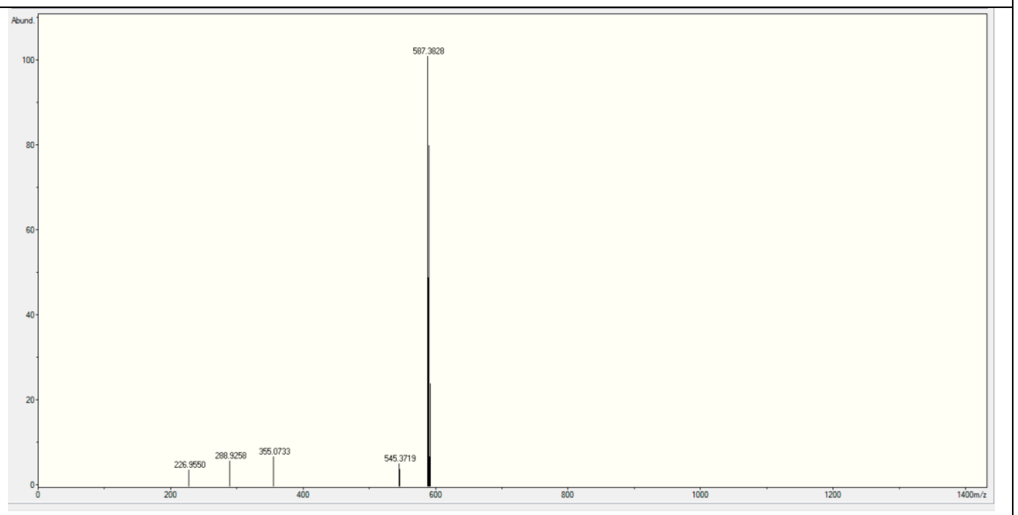

30.	3-acetoxy-3-0-methyl ester of glycyrrhetinic acid (AAK133)	oleanane	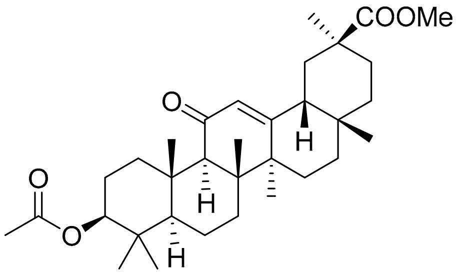 Chemical Formula: $C_{33}H_{50}O_5$ Exact Mass: 526.3658	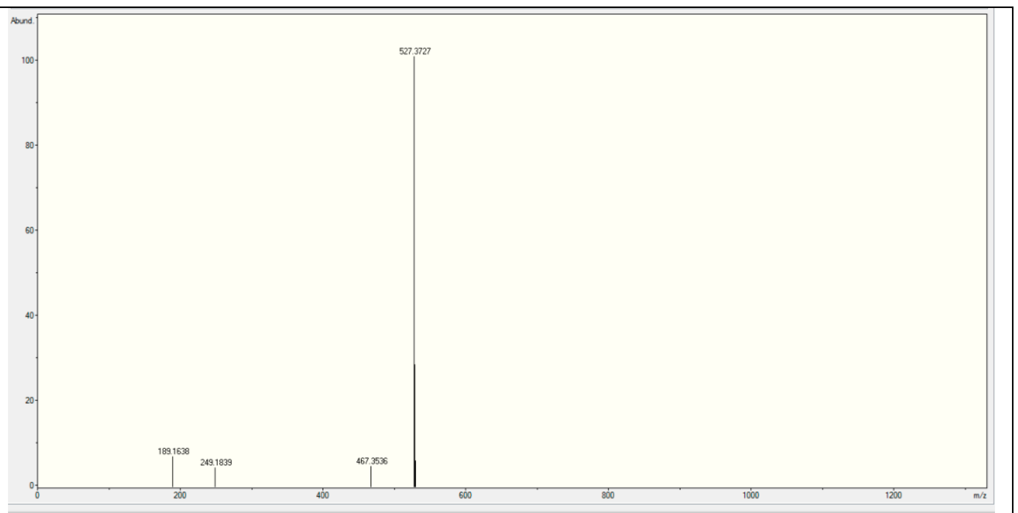
31.	Hydrazide of glycyrrhetinic acid (AAK112)	oleanane	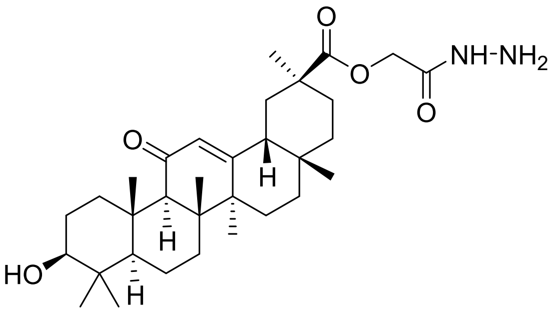 Chemical Formula: $C_{32}H_{50}N_2O_5$ Exact Mass: 542.3720	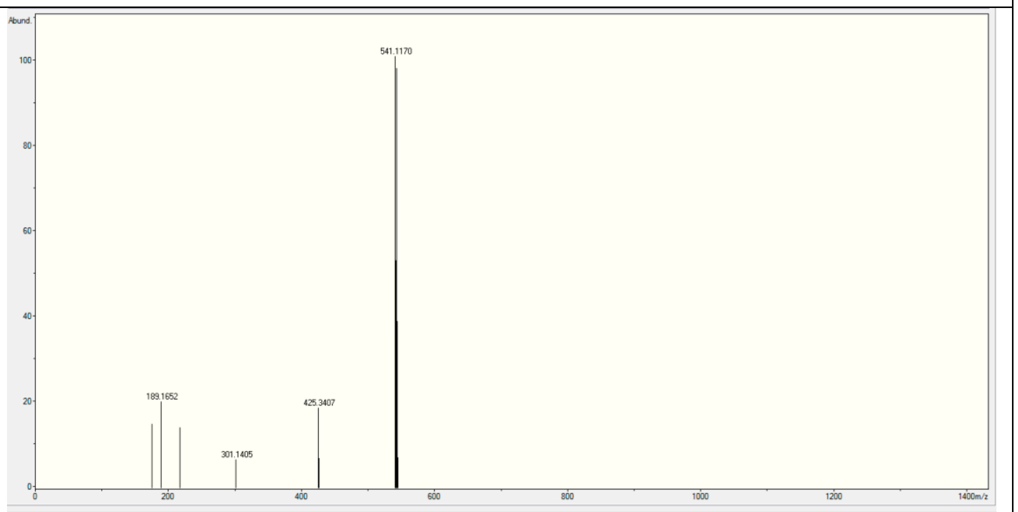
32.	Ethyl acetate ester of glycyrrhetinic acid (AAK141)	oleanane	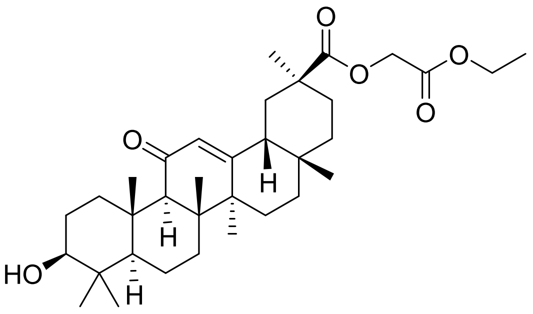 Chemical Formula: $C_{34}H_{52}O_6$ Exact Mass: 556.3764	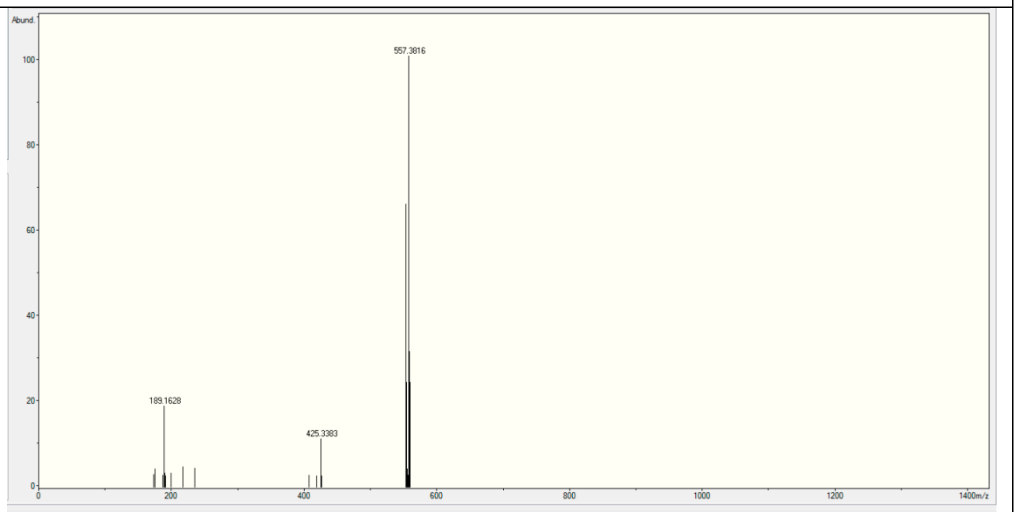
33.	Methyl acetate ester of glycyrrhetinic acid (AAK142)	oleanane	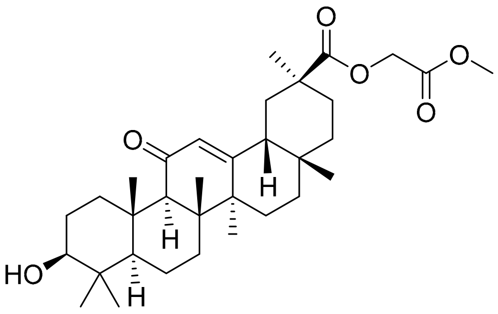 Chemical Formula: $C_{33}H_{50}O_6$ Exact Mass: 542.3607	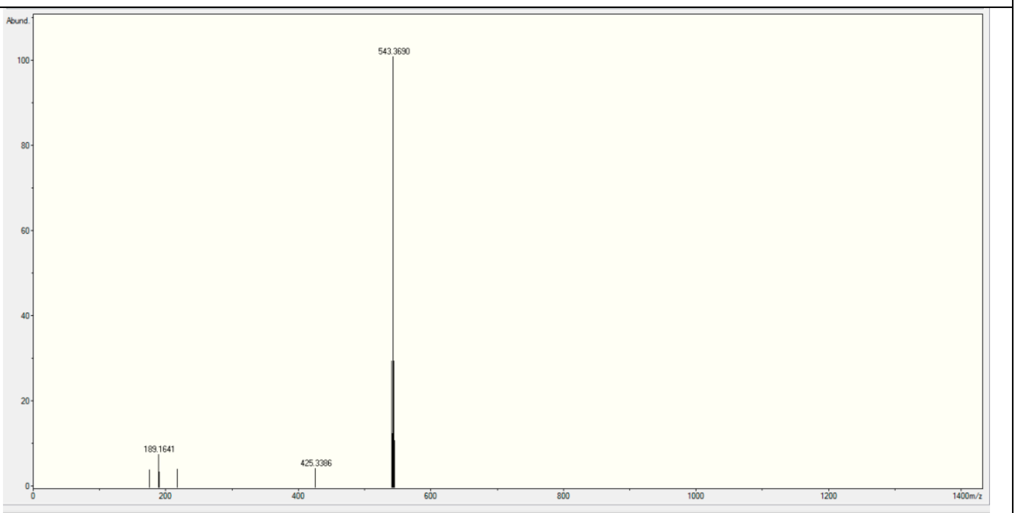
34.	Ethyl methyl sulfide ester of betulinic acid (AAK147)	Lupane	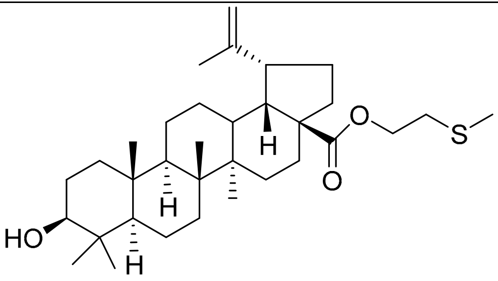 Chemical Formula: $C_{33}H_{54}O_3S$ Exact Mass: 530.3794	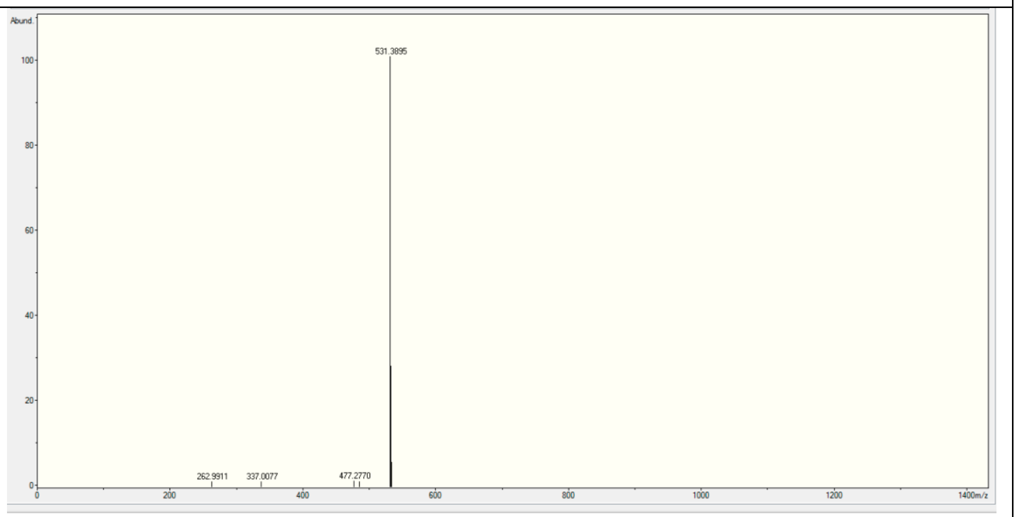

35.	Methyl acetate ester of betulinic acid<br>(AAK152)	Lupane	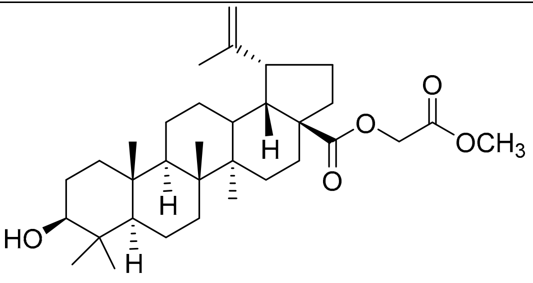<br>Chemical Formula: $C_{33}H_{52}O_5$<br>Exact Mass: 528.3815	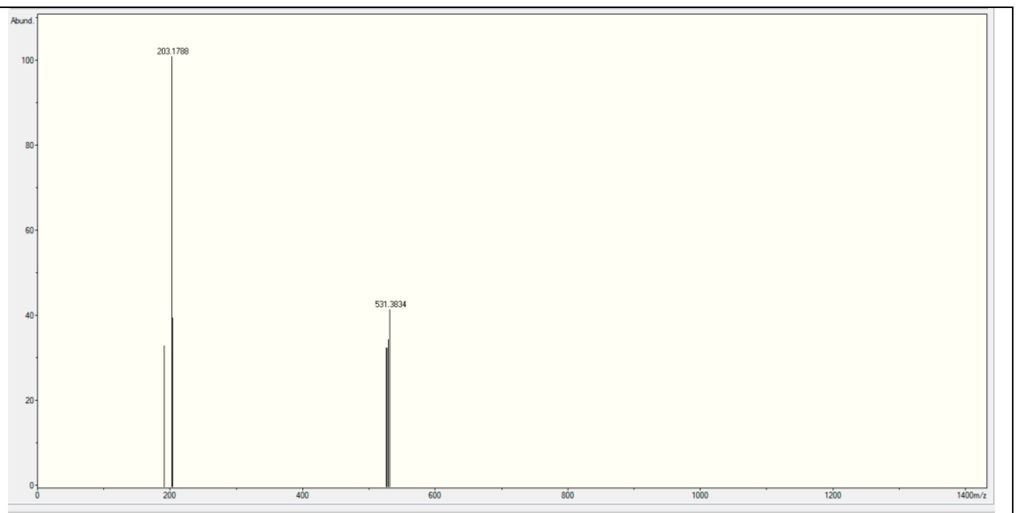
36.	Diethyl sulfide ester of betulinic acid<br>(AAK155)	Lupane	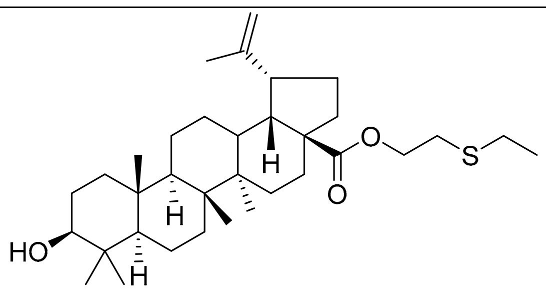<br>Chemical Formula: $C_{34}H_{56}O_3S$<br>Exact Mass: 544.3950	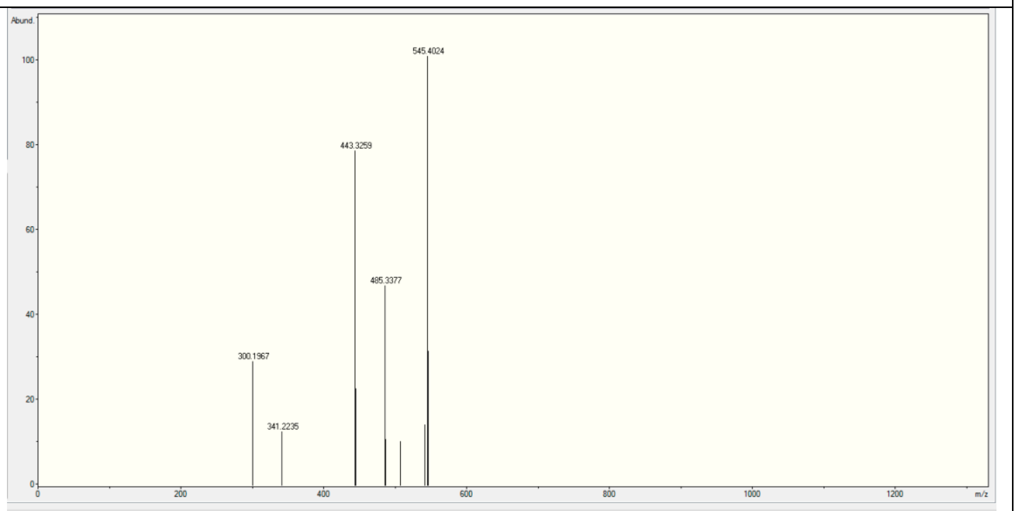
37.	Diethyl sulfide ester of ursolic acid<br>(AAK172)	Ursane	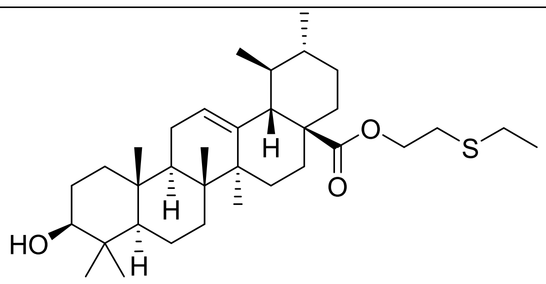<br>Chemical Formula: $C_{34}H_{56}O_3S$<br>Exact Mass: 544.3950	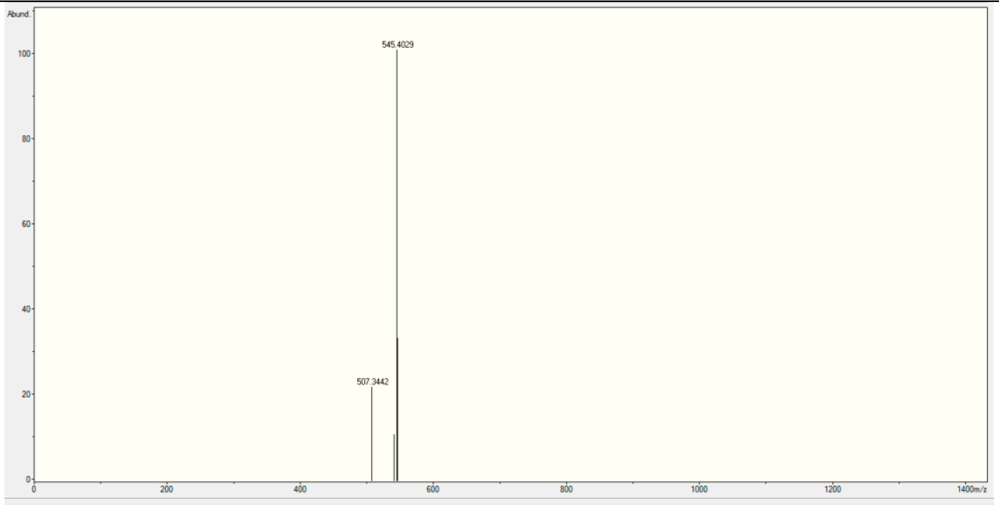
38.	Methyl acetate ester of oleanolic acid<br>(AAK160)	oleanane	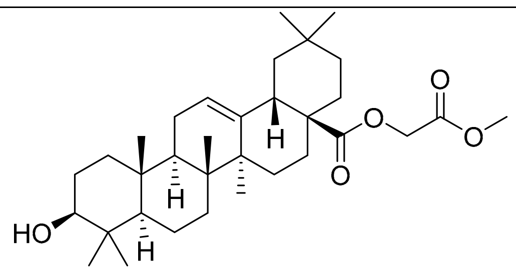<br>Chemical Formula: $C_{33}H_{52}O_5$<br>Exact Mass: 528.3815	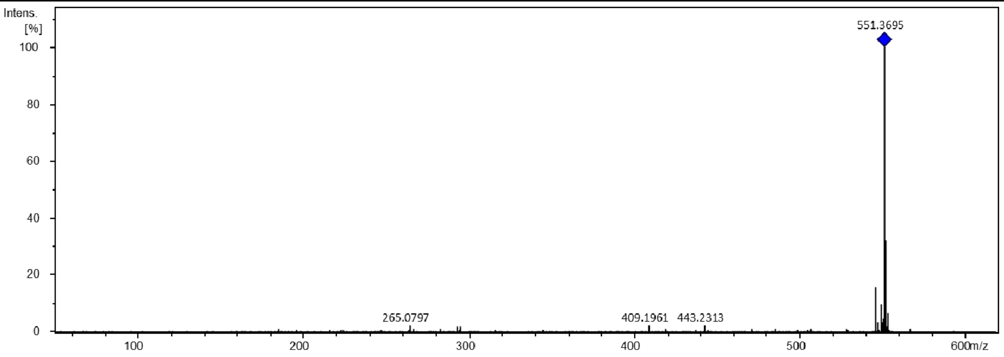
39.	2, 3, 23-triacetoxy derivative of asiatic acid<br>(AAK175)	Ursane	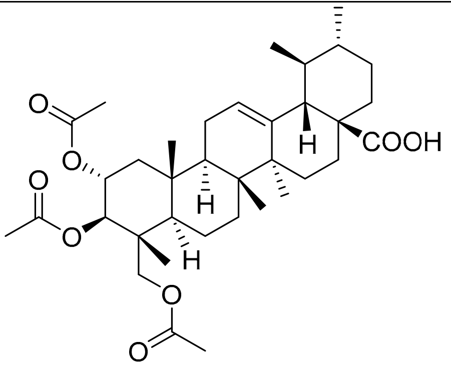<br>Chemical Formula: $C_{36}H_{54}O_8$<br>Exact Mass: 614.3819	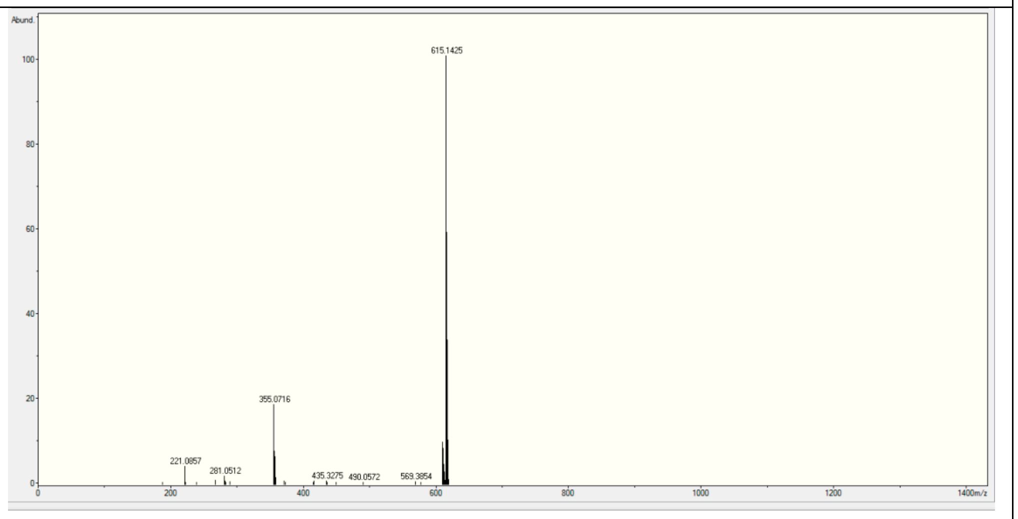

40.	Friedelin<br>(AAC150)	Friedelane	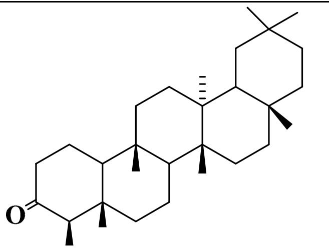<br>Chemical Formula: $C_{30}H_{50}O$<br>Exact Mass: 426.3862	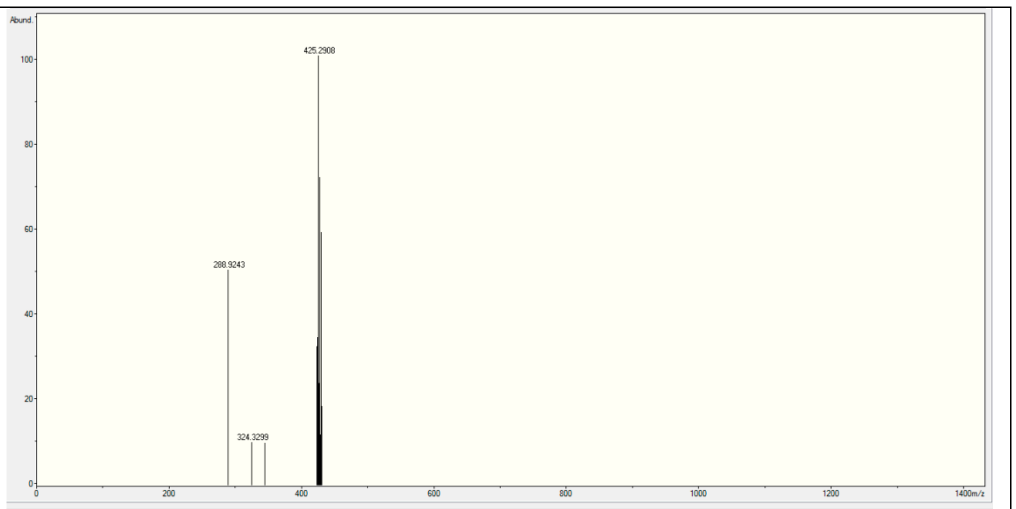
41.	2|A-Hydroxyursolic acid<br>(AAK095)	Ursane	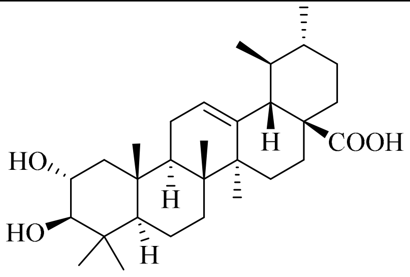<br>Chemical Formula: $C_{30}H_{48}O_4$<br>Exact Mass: 472.3553	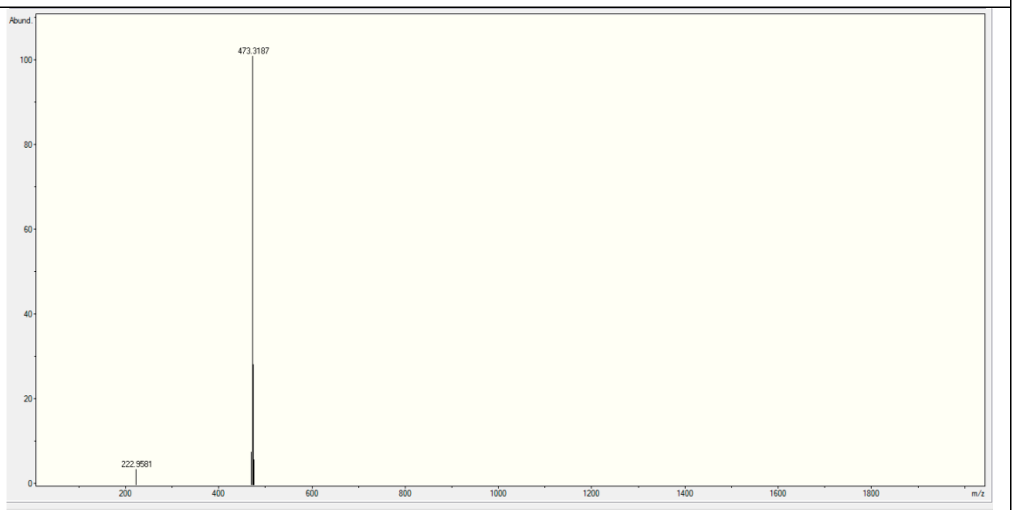
42.	beta-amyrin<br>(AAD671)	Oleanane	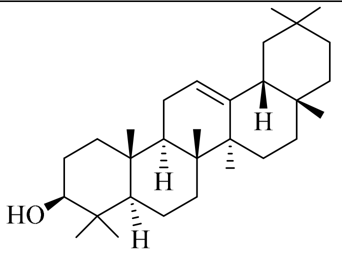<br>Chemical Formula: $C_{30}H_{50}O$<br>Exact Mass: 426.3862	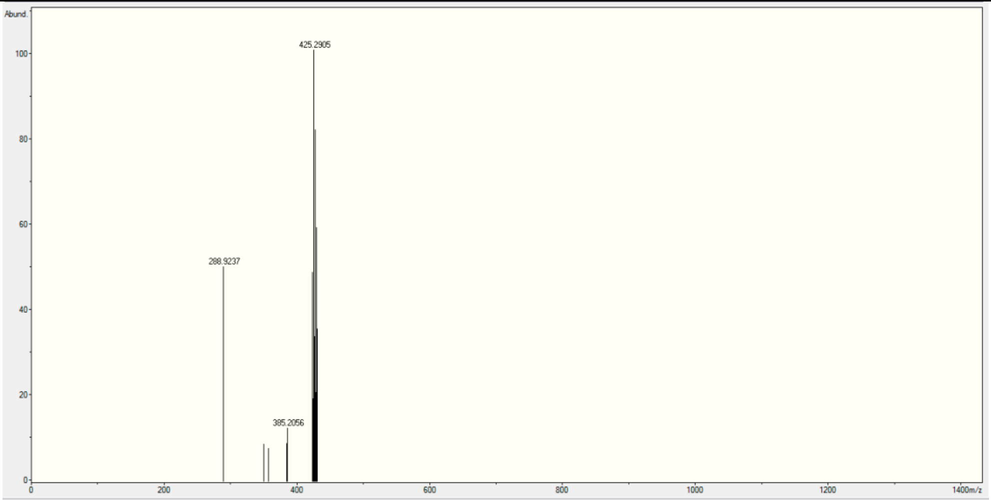
43.	3b-Hydroxy-27-p-E-coumaroyloxy-urs-12-en-28-oic acid<br>(AAC739)	Ursane	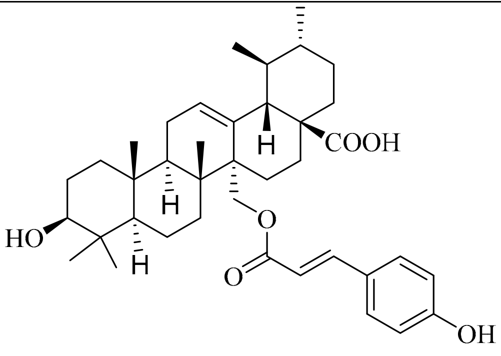<br>Chemical Formula: $C_{39}H_{54}O_6$<br>Exact Mass: 618.3920	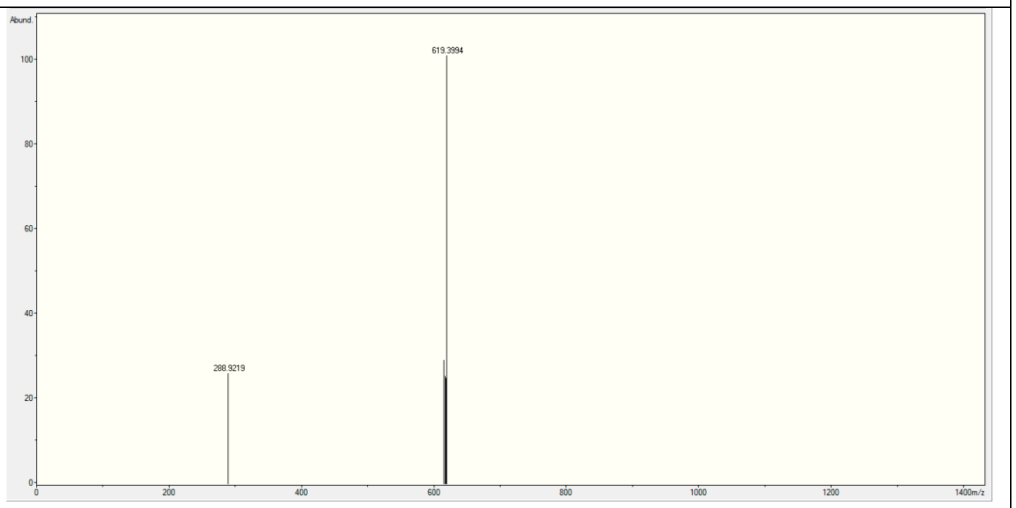
44.	Atriplicin<br>(AAC401)	oleanane	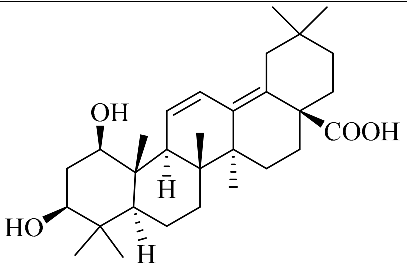<br>Chemical Formula: $C_{30}H_{46}O_4$<br>Exact Mass: 470.3396	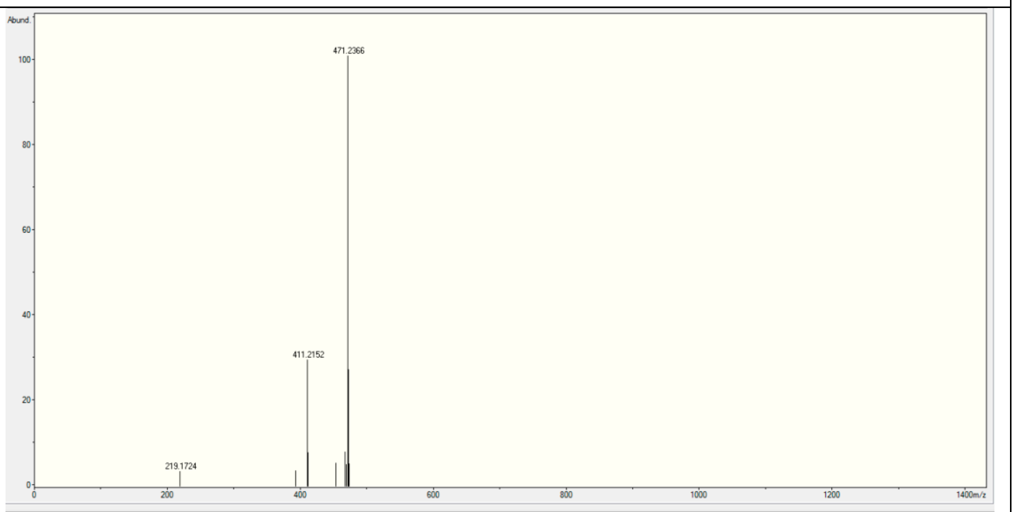
